# Transcriptome-Wide Effects of Sphingosine Kinases Knockdown in Metastatic Prostate and Breast Cancer Cells: Implications for Therapeutic Targeting

**DOI:** 10.3389/fphar.2019.00303

**Published:** 2019-03-27

**Authors:** Heba Alshaker, Qi Wang, Daniel Brewer, Dmitri Pchejetski

**Affiliations:** ^1^School of Medicine, University of East Anglia, Norwich, United Kingdom; ^2^Department of Pharmacology and Biomedical Sciences, Faculty of Pharmacy and Medical Sciences, University of Petra, Amman, Jordan; ^3^Earlham Institute, Norwich, United Kingdom

**Keywords:** sphingosine kinase, gene knockdown, DNA microarray, transcriptome, breast cancer, prostate cancer, molecular targets, targeted therapy

## Abstract

Sphingosine kinases 1 and 2 (SK1 and SK2) are proto-oncogenic isozymes expressed in many human tumors and associated with chemoresistance and poor prognosis. They are well-recognized therapy targets and their inhibition was shown to induce tumor volume reduction and chemosensitization in multiple cancer models. Oncogenic signaling is extremely complex and often cross-regulated. Designing molecular therapies and their combinations requires rational approaches to avoid redundant targeting or developing resistance. In this study, we have performed RNA transcriptome microarray analysis of two breast and two prostate metastatic cancer cell lines treated with siRNAs targeting SK1 or SK2. In prostate cancer cell lines SK1 knockdown (KD) has significantly changed expression of several genes including downregulation of *NSUN2, G3BP2* and upregulation of ETS1. SK2 KD also affected expression of multiple genes including downregulation of *CAPZA1 NSUN3* and *ADPGK* and upregulation of *VDAC1, IBTK, ETS1*, and *MKNK2*. Similarly, in breast cancer cells SK1 KD led to downregulation of *NSUN2, NFATC3, CDK2*, and *G3BP2* and upregulation of *GTF2B, TTC17*, and *RAB23*. SK2 KD in breast cancer cells has decreased expression of *ITGAV* and *CAPZA1* and increased expression of *GTF2B* and *ST13*. Gene-set enrichment analysis of known biochemical pathways showed that in prostate and breast cell lines SKs KD have altered multiple pathways. SK1 KD altered chromatin assembly, regulation of G1/S transition and mitosis, Wnt and MAP kinase signaling and cell motility. SK2 KD altered RAS protein signal transduction, regulation of MAP kinase and serine/threonine kinase activity, cell motility, small GTPase mediated signal transduction and phosphatidylinositol 3-kinase (PI3K) signaling. Through genome-wide microarray analysis, we have identified important molecular pathways affected by SK1 and SK2 KD. It appears that while KD of both genes leads to a decrease in individual pro-tumorigenic genes, there is a universal cellular response resulting in upregulation of several known pro-survival and pro-tumorigenic pathways such as MAPK, RAS, and PI3K, which may mediate cancer resistance to anti-SKs therapies. Our data point out to the potential advantage of certain molecular therapy combinations in targeting prostate and breast cancer. Further signaling studies are required to confirm the individual involvement of identified pathways.

## Introduction

Sphingosine kinases 1 and 2 (SK1 and SK2) are proto-oncogenic enzymes expressed in multiple human tumors (Alshaker et al., 2013). They convert proapoptotic lipid second messenger sphingosine to anti-apoptotic sphingosine-1-phosphate. SKs display different subcellular localization and tissue expression patterns. SK1 is a constitutively active cytosolic enzyme, that translocates to the plasma membrane upon phosphorylation at Ser225. SK2 is localized mainly in the nucleus where it has a role in DNA synthesis and histone H3 acetylation (Song et al., 2018).

There is compelling evidence that SK1 activation contributes to cancer progression and leads to oncogenic transformation, increased tumor growth and impairment of apoptosis (reviewed in Alshaker et al., 2013; White et al., 2016). SK1 is a tumor-associated enzyme: high levels of SK1 expression have been shown in various human tumors such as brain, breast, colon, lung, ovary, stomach, uterus, kidney, rectum, and small intestine, where they enhance tumor neovascularization and metastatic potential by promoting motility and invasion of cancer cells (Cuvillier, 2007; Shida et al., 2008; Pyne et al., 2012). High levels of SK1 expression or activity are associated with a poor prognosis in several human cancers, making it a key pathway in the search for targeted therapies (Pyne et al., 2012). SK2 is predominantly localized to cell organelles and its role in cell proliferation/apoptosis is less well studied. However, several studies showed a critical role of SK2 for epidermal growth factor-stimulated migration of breast cancer cells, growth of tumor xenografts and lung cancer chemoresistance (Song et al., 2018).

Multiple SK1 and SK2 inhibitors have been synthesized and assayed in different biological systems. Selective SK1 inhibitors such as SK1-I or SK-F have been demonstrated to efficiently induce apoptosis in cancer cells (Paugh et al., 2008; Alshaker et al., 2018). A sphingosine analog FTY720 was shown to inhibit SK1 and induce cancer cell apoptosis (Wang et al., 1999; Permpongkosol et al., 2002), chemo- and radiosensitization (Pchejetski et al., 2010; Alshaker et al., 2017; Wang et al., 2017). Several studies have indicated an anti-tumorigenic role of a specific SK2 inhibitor ABC294640 (Antoon et al., 2011; Beljanski et al., 2011; Song et al., 2018).

Oncogenic signaling pathways are complex. Intervention with a single pathway usually leads to multiple pathways being affected due to cross-regulation. Based on this rationale, identifying signaling “hubs” has become popular in molecular therapy design, hoping that by targeting one key molecule, multiple signaling pathways can be regulated. This, however, can lead to undesirable side effects. Furthermore, targeting only one pathway does not always lead to desired effects due to the presence of parallel signaling pathways leading to the same end-point, prompting the use of combined therapies. Care should be taken in the creation of combined therapeutic interventions utilizing compounds that could target independent or compensatory pathways and therefore have synergistic effects. The rationale for such combinations could be derived from studying the gene expression and pathways regulation.

In this study, we have used for the first time RNA transcriptome microarray technology to investigate the transcriptome-wide effects of SK1 and SK2 downregulation. This approach mimics the therapeutic targeting of SKs by inhibitors and allows mapping the affected/unaffected signaling cascades. Our data provide useful insight for creating more robust therapeutic combinations for cancer targeting.

## Materials and Methods

### Cell Culture and Reagents

Human breast cancer cell lines MDA-MB-231 and BT-549 and human prostate cancer cells lines PC-3 and DU145 were purchased from ATCC (Manassas, VA, United States), and maintained in RPMI with 10% FCS, 50 U/ml penicillin, 50 μg/ml streptomycin and 2 mM glutamine (Sigma-Aldrich, St. Louis, MO, United States). Cell lines were kept in culture for up to 30 passages. Cells were seeded to reach 70–80% confluence by the end of the treatment. All chemicals unless specified were from Sigma Aldrich (Poole, United Kingdom).

### RNA Interference

Cells were seeded at a density to reach 30–50% confluence by the day of transfection. Cells were transfected as described previously (Alshaker et al., 2014, 2015) using siRNA directed against SK1 and SK2 as pooled four independent sequences and non-targeting siRNA as a negative control combined with HiPerFect (Qiagen, West Sussex, United Kingdom). Optimal knockdown (KD) was obtained 72 h post-transfection and verified by qRT-PCR.

### RNA Extraction, cDNA Synthesis and qRT-PCR

Isolation of total RNA from cancer cells was performed using the RNeasy Mini kit (Qiagen, Valencia, CA, United States) as per manufacturer’s instructions. RNA quantity and purity was measured using a NanoDrop 2000c Spectrophotometer (Thermo Fisher Scientific, Loughborough, United Kingdom). Reverse transcription was performed using Precision nanoScript^TM^ Reverse transcription kit (PrimerDesign Ltd., Southampton, United Kingdom). qRT-PCR was done as already described (Alshaker et al., 2014, 2017). Ct values were exported and analyzed using qbase software (Biogazelle NV, Zwijnaarde, Belgium).

### RNA Microarray

RNA was normalized to an input amount of 99.9 ng and underwent reverse transcription. sscDNA was purified using magnetic beads and fragmented using UDG. Fragmented sample was hybridized to Affymetrix Clariom S human arrays at 45°C overnight. Stained arrays are scanned to generate intensity data. All reagent kits and arrays were purchased from Thermo Fisher Scientific, Loughborough, United Kingdom.

### Sphingosine Kinase Activity

Sphingosine kinases 1 and 2 assay was performed using radiolabeling as previously described (Pchejetski et al., 2011; Alshaker et al., 2014, 2015), in conditions favoring either SK1 or SK2 activity (Liu et al., 2000).

### Statistical Analysis

qRT-PCR data are presented as the mean values of at least three independent experiments normalized to control ± standard error of the mean (SEM) calculated using GraphPad Prism. Statistical significance between two groups was conducted by unpaired Student’s *t*-test. *P*-value of <0.05 is considered statistically significant.

DNA microarray analyses were performed in R version 3.4.3. Gene-level signal estimates were derived from CEL files generated from Affymetrix Clariom S human arrays using the multi-array analysis (RMA) algorithm (Irizarry et al., 2003) implemented in Bioconductor package oligo version 1.42.0 (Carvalho and Irizarry, 2010). Differential expression analysis was performed using the *limma* version 3.34.4: linear models were determined for each transcript cluster (gene) and an estimate for the global variance calculated by an empirical Bayes approach (Smyth, 2004). A moderated *t*-statistic was computed for each transcript cluster with the resulting *p*-values adjusted for multiple testing using Benjamini and Hochberg’s method to control the false discovery rate. Those transcript clusters with an adjusted *p*-value less than 0.05 were considered to be significantly differentially expressed between the two groups.

Gene ontology biological process and hallmarks of cancer gene sets were tested in gene set enrichment analysis using the clusterProfiler package, version 3.6 (Yu et al., 2012). The *t*-statistic generated in the differential expression analyses was used as the metric, with all entrez genes as the background and a cut-off *p*-value of 0.05 after multiple testing using the false discovery rate.

## Results

**FIGURE 1:**
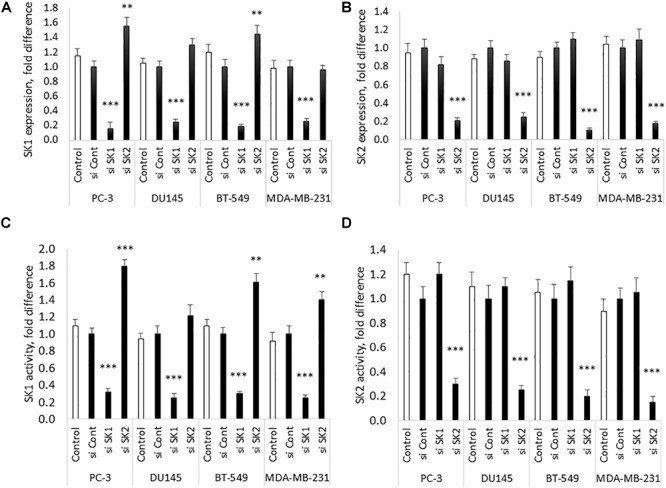
SK1/SK2 knockdown in prostate and breast cancer cell lines. Human breast cancer cell lines MDA-MB-231 and BT-549 and human prostate cancer cells lines PC-3 and DU145 were transfected with SK1 and SK2 siRNA and gene knockdown was assessed by qRT-PCR **(A,B)**. SK1 and SK2 activity was assessed using radiolabeling assay **(C,D)**. Data mean values of at least three independent experiments normalized to control ± SEM. ^∗∗^*p* < 0.01, ^∗∗∗^*p* < 0.001.

Chemotherapy and molecular therapy are currently used in treatment of locally advanced and metastatic prostate and breast cancers. Among those cancers, androgen-independent prostate tumors and triple negative breast tumors are considered the most aggressive form of the disease. As a model of those diseases, we have chosen two metastatic androgen-insensitive prostate cancer cells lines PC-3 and DU145 and two metastatic triple negative breast cancer cell lines MBA-MB-231 and BT-549. SK1 and SK2 downregulation was achieved by transfection with four pooled sequences siRNA (Qiagen) for 72 h and verified using qRT-PCR (Figure 1). It is interesting to note that in PC-3 and BT-549 cells KD of SK1 caused a significant increase in SK2 expression (55 and 45%, respectively), however this effect was not present in MDA-MB-231 and DU145 cells (Figure 1). These results were confirmed on the enzyme level using SK1 and SK2 activity (Figure 1).

**FIGURE 2:**
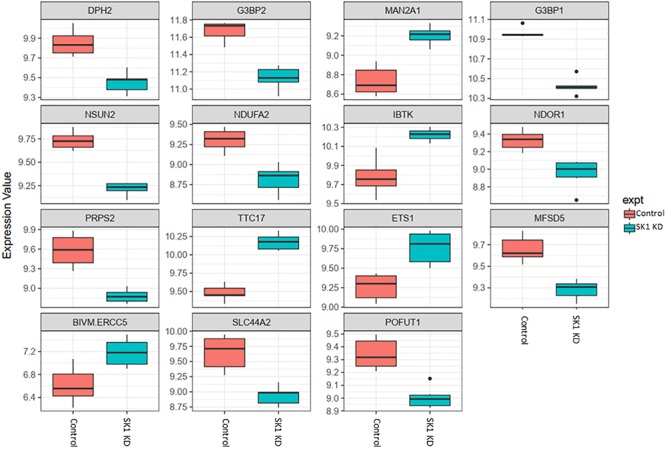
Altered gene expression in response to SK1 knockdown (KD) in prostate cancer cell lines. Human prostate cancer cells lines PC-3 and DU145 were transfected with SK1 siRNA and Affymetrix Clariom S human array was performed as described in materials and methods. Differential expression analysis was performed using the *limma* version 3.34.4: linear models were determined for each transcript cluster (gene) and an estimate for the global variance calculated by an empirical Bayes approach. A moderated *t*-statistic was computed for each transcript cluster with the resulting *p*-values adjusted for multiple testing using Benjamini and Hochberg’s method to control the false discovery rate. Those transcript clusters with an adjusted *p*-value less than 0.05 were considered to be significantly differentially expressed between the two groups; *bars*, SEM.

**FIGURE 3:**
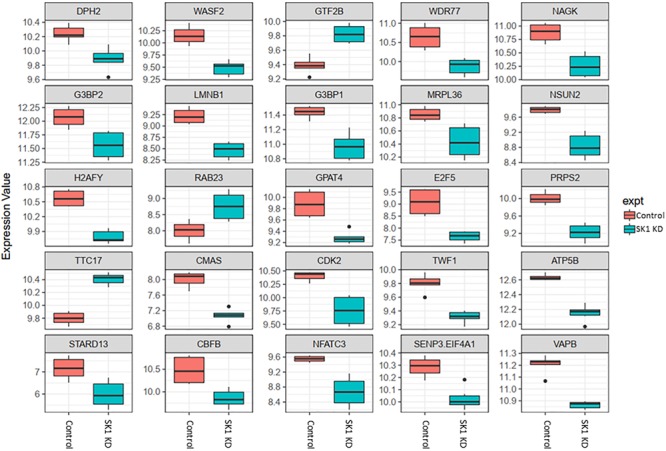
Altered gene expression in response to SK1 knockdown (KD) in breast cancer cell lines. Human breast cancer cell lines MDA-MB-231 and BT-549 were transfected with SK1 siRNA and Affymetrix Clariom S human array was performed as described in materials and methods. Differential expression analysis was performed using the *limma* version 3.34.4: linear models were determined for each transcript cluster (gene) and an estimate for the global variance calculated by an empirical Bayes approach. A moderated *t*-statistic was computed for each transcript cluster with the resulting *p*-values adjusted for multiple testing using Benjamini and Hochberg’s method to control the false discovery rate. Those transcript clusters with an adjusted *p*-value less than 0.05 were considered to be significantly differentially expressed between the two groups; *bars*, SEM.

**FIGURE 4:**
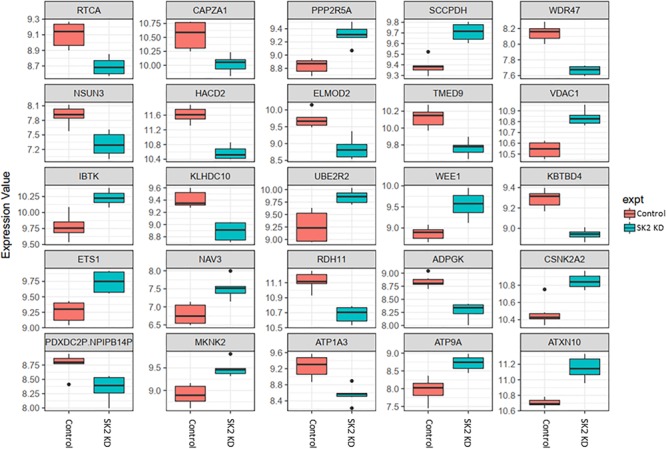
Altered gene expression in response to SK2 knockdown (KD) in prostate cancer cell lines. Human prostate cancer cells lines PC-3 and DU145 were transfected with SK2 siRNA and Affymetrix Clariom S human array was performed as described in materials and methods. Differential expression analysis was performed using the *limma* version 3.34.4: linear models were determined for each transcript cluster (gene) and an estimate for the global variance calculated by an empirical Bayes approach. A moderated *t*-statistic was computed for each transcript cluster with the resulting *p*-values adjusted for multiple testing using Benjamini and Hochberg’s method to control the false discovery rate. Those transcript clusters with an adjusted *p*-value less than 0.05 were considered to be significantly differentially expressed between the two groups; *bars*, SEM.

**FIGURE 5:**
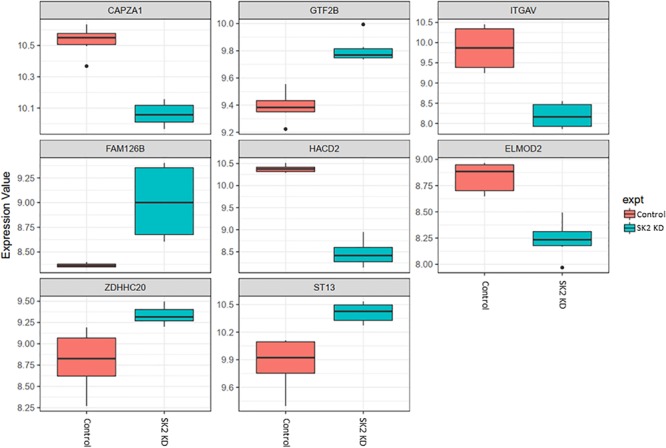
Altered gene expression in response to SK2 knockdown (KD) in breast cancer cell lines. Human breast cancer cell lines MDA-MB-231 and BT-549 were transfected with SK2 siRNA and Affymetrix Clariom S human array was performed as described in materials and methods. Differential expression analysis was performed using the *limma* version 3.34.4: linear models were determined for each transcript cluster (gene) and an estimate for the global variance calculated by an empirical Bayes approach. A moderated *t*-statistic was computed for each transcript cluster with the resulting *p*-values adjusted for multiple testing using Benjamini and Hochberg’s method to control the false discovery rate. Those transcript clusters with an adjusted *p*-value less than 0.05 were considered to be significantly differentially expressed between the two groups; *bars*, SEM.

DNA CHIP analysis was performed on all samples in triplicate using Affymetrix Clariom S microarray. Genes were determined to be significantly differentially expressed using a moderated *t*-test (*p* (0.05; Benjamin Hochberg multiple testing correction applied). SK1 and SK2 regulated genes exhibited tissue specific differences. As expected, SK1 and SK2 were shown to be down-regulated by respective siRNA pools (for clarity, these effects are not shown in Figure 2–5).

In prostate cancer cells SK1 KD induced downregulation of expression of ten genes and upregulation of five genes (Figure 2, only genes identically regulated in both cell lines are shown). Among the genes downregulated by SK1 were G3BP stress granule assembly factors G3BP1 and G3BP2 [involved in nuclear factor kappa B (NFκB), RAS and Wnt signaling], phosphoribosyl pyrophosphate synthetase 2 (PRPS2), involved in multiple biosynthesis pathways like purine synthesis, NOP2/Sun RNA methyltransferase family member 2 (NSUN2) which regulates mRNA translation and NADH:ubiquinone oxidoreductase subunit A2 (NDUFA2), a subunit of complex I of the respiratory chain. SK1 KD increased expression of IBTK (inhibitor of Bruton tyrosine kinase) and notably ETS1 (ETS proto-oncogene 1, transcription factor).

In breast cancer cells SK1 KD (Figure 3) has decreased the expression of E2F5 (E2F transcription factor 5), NSUN2, nuclear factor of activated T-cells 3 (NFATC3) which regulates transcription, PRPS2, cyclin dependent kinase 2 (CDK2), known regulator in the cell cycle, G3BP stress granule assembly factors G3BP1 and G3BP2, vesicle-associated membrane-protein-associated protein B (VAPB) involved in vesicle trafficking and N-acetylglucosamine kinase (NAGK), involved in metabolism. SK1 KD has notably increased expression of RAB23 (encodes a small GTPase of the RAS oncogene superfamily).

In prostate cells, SK2 KD affected expression of 25 genes (Figure 4) including downregulation of capping actin protein of muscle Z-line alpha subunit 1 (CAPZA1), involved in inhibition of autophagy and epithelial-mesenchymal transition (EMT), NSUN3 (NOP2/Sun RNA methyltransferase family member 3, mRNA translation), ADP dependent glucokinase (ADPGK), involved in EMT in cancer cells and kelch domain containing 10 (KLHDC10), which regulates oxidative stress. SK2 KD increased expression of voltage dependent anion channel 1 (VDAC1) oncogene, regulator of mammalian target of rapamycin (mTOR), inhibitor of Bruton tyrosine kinase (IBTK), antiproliferative, ataxin 10 (ATXN10) which activates the RAS-MAP kinase pathway, ETS proto-oncogene 1 (ETS1), transcription factor, ubiquitin conjugating enzyme E2 R2 (UBE2R2) involved in ubiquitination and cell differentiation, MAP kinase interacting serine/threonine kinase 2 (MKNK2), protein phosphatase 2 regulatory subunit B′alpha (PPP2R5A) and WEE1 G2 checkpoint kinase (WEE1), cell cycle regulator.

On the other hand, SK2 KD in breast cancer (Figure 5) has downregulated expression of integrin subunit alpha V (ITGAV), belongs to the integrin alpha chain family which may regulate angiogenesis and cancer progression and capping actin protein of muscle Z-line alpha subunit 1 (CAPZA1), regulates growth of the actin filament and increased expression of general transcription factor IIB (GTF2B), zinc finger DHHC-type containing 20 (ZDHHC20) and ST13, Hsp70 interacting protein, involved in the assembly process of glucocorticoid receptor.

**FIGURE 6:**
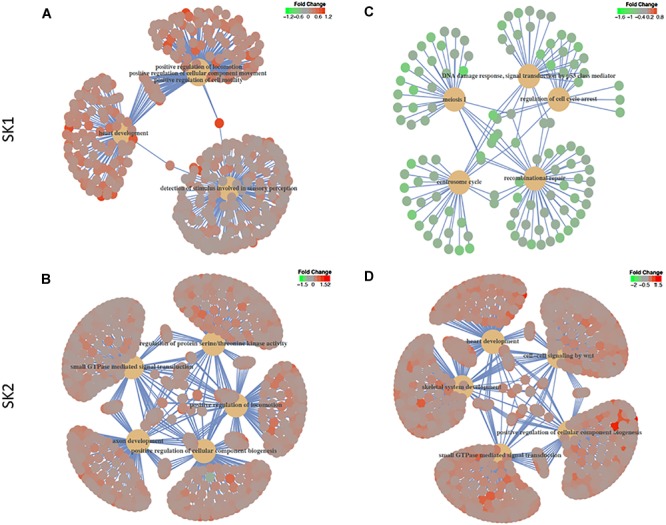
Gene-set enrichment analysis of gene ontology biological process enrichment pathways. Human prostate cancer cells lines PC-3 and DU145 **(A,B)** and human breast cancer cell lines MDA-MB-231 and BT-549 **(C,D)** were transfected with SK1 **(A,C)** and SK2 **(B,D)** siRNA and Affymetrix Clariom S human array was performed as described in materials and methods. Gene ontology biological process and hallmarks of cancer gene sets were tested in gene set enrichment analysis using the clusterProfiler package, version 3.6. The *t*-statistic generated in the differential expression analyses was used as the metric, with all Entrez Genes as the background and a cut-off *p*-value of 0.05 after multiple testing using the false discovery rate.

We have then performed a functional analysis of identified genes for the gene-set enrichment of known biochemical pathways upon SK1 and 2 KD. We have investigated the changes for gene ontology biological process pathways (Figure 6 and Supplementary Tables S1–S4) and hallmarks of cancer gene sets (Tables 1–4). SK1 and SK2 KD had different effect on pathway enrichment which was also cell line dependent, although there were several common pathways shared between both cell lines and both SKs. Gene-set enrichment analysis of gene ontology biological process enrichment pathways showed that in prostate cancer cells SK1 KD has induced downregulation of pathways linked with DNA conformation change, chromatin assembly and regulation of G1/S transition of mitotic cell cycle and notably enrichment of Wnt signaling pathway, cell motility and MAP kinase activity (Supplementary Table S1, examples of linked pathways shown in Figure 6). In breast cancer cells SK1 KD has induced a downregulation of pathways responsible for DNA replication and repair, cell cycle (G1/S), p53, enrichment in Wnt signaling and vesicle-mediated transport pathways (Supplementary Table S2, examples of linked pathways shown in Figure 6). In prostate cancer cells, SK2 KD has enriched RAS protein signal transduction, MAP kinase, protein serine/threonine kinase, cell motility, small GTPase and phosphatidylinositol 3-kinase signaling pathways (Supplementary Table S3, examples of linked pathways shown in Figure 6) while in breast cancer cells it induced Wnt signaling, small GTPase mediated signal transduction, MAP kinase, endosomal transport and RAS protein signal transduction pathways and downregulated DNA repair pathway (Supplementary Table S4, examples of linked pathways shown in Figure 6).

**Table 1:** Gene-set enrichment analysis of hallmark of cancer enrichment pathways in response to SK1 KD in breast cancer cells.

Description	Enrichment score	*p*-value
HALLMARK_E2F_TARGETS	–0.58603	0.001923
HALLMARK_G2M_CHECKPOINT	–0.45283	0.001905
HALLMARK_MYC_TARGETS_V1	–0.40462	0.001923
HALLMARK_UV_RESPONSE_UP -0.32927	0.012121
HALLMARK_INFLAMMATORY_ RESPONSE	0.285129	0.026971
HALLMARK_KRAS_SIGNALING_UP	0.287628	0.024896
HALLMARK_IL2_STAT5_SIGNALING	0.296696	0.010526
HALLMARK_APOPTOSIS	0.300881	0.026
HALLMARK_GLYCOLYSIS	0.30635	0.004149
HALLMARK_TNFA_SIGNALING_VIA_ NFKB	0.314365	0.004211
HALLMARK_COMPLEMENT	0.32964	0.004211
HALLMARK_APICAL_JUNCTION	0.340443	0.002075
HALLMARK_MYOGENESIS	0.34409	0.002105
HALLMARK_P53_PATHWAY	0.354588	0.002096
HALLMARK_HYPOXIA	0.355858	0.002105
HALLMARK_FATTY_ACID_ METABOLISM	0.373058	0.003984
HALLMARK_XENOBIOTIC_ METABOLISM	0.374965	0.002075
HALLMARK_ADIPOGENESIS	0.381432	0.002105
HALLMARK_COAGULATION	0.404393	0.001996
HALLMARK_INTERFERON_GAMMA_ RESPONSE	0.4096	0.00211
HALLMARK_EPITHELIAL_ MESENCHYMAL_TRANSITION	0.414758	0.002105
HALLMARK_UV_RESPONSE_DN	0.437977	0.001976

**Table 2:** Gene-set enrichment analysis of hallmark of cancer enrichment pathways in response to SK1 KD in prostate cancer cells.

Description	Enrichment score	*p*-value
HALLMARK_UV_RESPONSE_UP	–0.402737052	0.0027
HALLMARK_E2F_TARGETS	–0.382852104	0.0028
HALLMARK_G2M_CHECKPOINT	0.373919221	0.0028
HALLMARK_P53_PATHWAY	–0.311816056	0.0028
HALLMARK_XENOBIOTIC_METABOLISM	–0.31162798	0.0028
HALLMARK_INTERFERON_GAMMA_ RESPONSE	–0.308977776	0.0028
HALLMARK_FATTY_ACID_METABOLISM	–0.290319736	0.0134
HALLMARK_ADIPOGENESIS	–0.290165971	0.0057
HALLMARK_OXIDATIVE_ PHOSPHORYLATION	–0.273082408	0.0113
HALLMARK_COMPLEMENT	0.283316334	0.0246
HALLMARK_HYPOXIA	0.291083127	0.0185
HALLMARK_IL2_STAT5_SIGNALING	0.295293643	0.0154
HALLMARK_TNFA_SIGNALING_VIA_ NFKB	0.317881573	0.0077
HALLMARK_MITOTIC_SPINDLE	0.333397383	0.0046
HALLMARK_KRAS_SIGNALING_UP	0.367158279	0.0015
HALLMARK_EPITHELIAL_ MESENCHYMAL_TRANSITION	0.440097468	0.0015

**Table 3:** Gene-set enrichment analysis of hallmark of cancer enrichment pathways in response to SK2 KD in breast cancer cells.

Description	Enrichment score	*p*-value
HALLMARK_INTERFERON_GAMMA_ RESPONSE	–0.389698297	0.0043
HALLMARK_ALLOGRAFT_REJECTION	–0.339536274	0.0043
HALLMARK_KRAS_SIGNALING_DN	–0.28487173	0.0042
HALLMARK_E2F_TARGETS	–0.278165758	0.0085
HALLMARK_G2M_CHECKPOINT	–0.270306348	0.0129
HALLMARK_GLYCOLYSIS	0.317865223	0.0222
HALLMARK_FATTY_ACID_METABOLISM	0.326776858	0.0214
HALLMARK_MITOTIC_SPINDLE	0.348623881	0.0052
HALLMARK_XENOBIOTIC_METABOLISM	0.356201125	0.0013
HALLMARK_HYPOXIA	0.356508267	0.0013
HALLMARK_APICAL_JUNCTION	0.356988995	0.0013
HALLMARK_EPITHELIAL_ MESENCHYMAL_TRANSITION	0.373510215	0.0013
HALLMARK_ADIPOGENESIS	0.382867908	0.0013
HALLMARK_HEME_METABOLISM	0.390882683	0.0013
HALLMARK_P53_PATHWAY	0.424168593	0.0013
HALLMARK_MYOGENESIS	0.429404077	0.0013
HALLMARK_UV_RESPONSE_DN	0.445254468	0.0014

**Table 4:** Gene-set enrichment analysis of hallmark of cancer enrichment pathways in response to SK2 KD in prostate cancer cells.

Description	Enrichment score	*p*-value
HALLMARK_INTERFERON_GAMMA_ RESPONSE	–0.45775	0.003021148
HALLMARK_UV_RESPONSE_UP	–0.35239	0.002881844
HALLMARK_FATTY_ACID_ METABOLISM	–0.30608	0.00877193
HALLMARK_HEME_METABOLISM	0.3308	0.004470939
HALLMARK_E2F_TARGETS	0.33106	0.004451039
HALLMARK_G2M_CHECKPOINT	0.34475	0.001501502
HALLMARK_UV_RESPONSE_DN	0.3727	0.003120125
HALLMARK_MITOTIC_SPINDLE	0.47889	0.00148368

Gene-set enrichment analysis of hallmarks of cancer pathways has shown several important pathways regulated by SK1/SK2 KD. SK1 KD in breast cancer cell lines downregulated Myc, G2/M cell cycle and E2F pathways (Table 1) and upregulated KRAS, IL2/signal transducer and activator of transcription (STAT)5 and tumor necrosis factor (TNF)/NFκB pathways. Prostate cancer cell lines similarly had G2/M, E2F and additionally p53 pathways downregulated, and KRAS, IL2/STAT5, TNF/NFκB and additionally EMT pathways upregulated by SK1 KD (Table 2).

Sphingosine kinase 2 KD had similar effects to SK1 in breast cancer cells downregulating KRAS, G2/M and E2F pathways and upregulating EMT and p53 pathways (Table 3). On the contrary, in prostate cancer cells SK2 KD has upregulated G2/M and E2F pathways (Table 4).

## Discussion

In the last decade SK inhibitors have shown significant potential for cancer treatment. There are dozens of papers proving their antitumour efficacy *in vitro* and *in vivo* (reviewed in Alshaker et al., 2013) and two molecules are already in clinical trials: SK1 inhibitor phenoxodiol (Veyonda) for prostate cancer, non-small cell lung cancer and sarcoma; and SK2 inhibitor ABC294640 (opaganib) for advanced solid tumors and multiple myeloma. These inhibitors are often proposed to be used as “sensitisers” to chemo- and radiotherapy and can be used as free drugs or in nanoparticle settings (Alshaker et al., 2017; Wang et al., 2017; Yee et al., 2017). Their specificity has significantly increased with the recent discovery of SK1 structure (Wang et al., 2013) and the use of computer modeling methods (Alshaker et al., 2018).

Cancer progression is mediated by multiple mutations and involves activation of a wide variety of signaling pathways, many of which are cross-regulated or lead to similar downstream events (reviewed in Garland, 2017). For example, in cancer cell, a mutation in receptor tyrosine kinase can activate multiple signaling pathways and subsequent transcription factors leading to gene expression, while each of these pathways can be also mutated or activated independently, creating a highly complex web of signaling. The typical molecular targeting therapy approach is to block these pathways (e.g., tyrosine kinases, mTOR, MAPK, PARP, CDK, etc.) with specific inhibitors. Aside from few cases (such as BCR-Abl), where one major mutation is responsible for cancer progression, it appears that switching off one pathway is usually insufficient to completely block cancer cell growth and induce cell death. Ordinarily, targeted cancer monotherapy can end up with bypass mechanisms. Resistant clones of cancer cells evolve that can compensate for the switched off pathway by upregulating other independent pathways.

Several approaches can be used to circumvent this phenomenon. First, improved drug delivery may allow achieving higher drug concentrations in the tumor leading to higher efficacy. Second, the employment of several combined targeted or non-targeted therapies or agents that interfere with multiple cell-signaling pathways may allow making multiple hits on the cell proliferation machinery. Finally, a combination of these approaches can possibly provide a significant benefit both in terms of efficacy and reducing side effects (Alshaker et al., 2017; Wang et al., 2017).

When designing the successful drug combinations, one may consider which pathways are implicated in proliferation and chemoresistance in the target system, as well as the known crosstalk between these pathways. For example, if a pro-survival pathway A activates a pro-survival pathway B, but doesn’t affect the pro-survival pathway C, targeting both A and B is redundant while targeting A and C may have synergistic therapeutic effect.

We have conducted this study to identify which signaling pathways are universally or tissue specifically regulated by SK1 and SK2 in prostate and breast cancers. More importantly, we questioned which common cell proliferation pathways are upregulated as compensatory mechanisms and may be responsible for resistance to anti-SK therapies. Considering that SK inhibitors may reach clinic in near future, this knowledge would allow us to hypothesize which combinational therapies may have synergistic effects.

Across all four cell lines investigated SK1 KD downregulated expression of NSUN2 (mRNA translation), PRPS2 (purine synthesis) and G3BP1,2 (NFκB, RAS, and Wnt signaling) (Figure 2, 3). Other notable genes decreased by SK1 KD were NDUFA2 in prostate and E2F5, NAGK, VAPB, NFATC3, CDK2 in breast cancer cell lines. SK2 KD universally downregulated expression of CAPZA1 (inhibition of autophagy and EMT) and also decreased expression of NSUN3, ADPGK and KLHDC10 in prostate and ITGAV in breast cancer cell lines (Figure 4, 5). From these data it appears that SK targeting carries a significant antiproliferative effect through downregulation of expression of several genes which are relevant for cell survival and division. However, individually SK1 and SK2 KD have led to upregulation of multiple genes (IBTK, GTF2B, VDAC1, GTF2B, ETS1, TTC17, IBTK, ZDHHC20, RAB23, ATXN10, ST13, ETS1, PPP2R5A, MKNK2, UBE2R2, WEE1) that regulate transcription, cell cycle, EMT, cell motility, serine/threonine kinases, small GTPases, phosphatidylinositol 3-kinase (PI3K), HSP70, Wnt, mTOR, RAS and MAPK signaling pathways (Jiang-Hua et al., 2014; Hu et al., 2018; Liu et al., 2018). It is very likely that these pathways represent a cellular “compensatory” response and may be potentially contributing to SK-inhibitor resistance.

**FIGURE 7:**
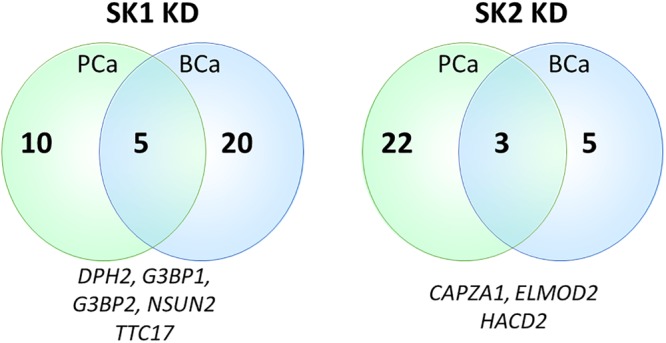
Venn diagram of genes regulated by SK1/SK2 knockdown (KD) in prostate and breast cell lines. Human breast cancer cell lines MDA-MB-231 and BT-549 and prostate cancer cells lines PC-3 and DU145 were transfected with SK1 or SK2 siRNA and Affymetrix Clariom S human array was performed as described in materials and methods. Numbers of significantly regulated genes common to prostate or breast cell lines are shown in outer semicircles and the number of genes universally regulated by SK1 or SK2 KD in all cell lines is shown in the middle. These universal genes are listed below the circles.

Sphingosine kinases 1 and 2 KD has up- and downregulated a significant number of genes in individual cell lines (Supplementary Table S5). This number was reduced to 15 and 25 genes for SK1 and SK2 KD, respectively, in prostate cells and 25 and 8 genes for SK1 and SK2 KD, respectively, in breast cancer cells (Figure 2–5, 7). There was some concordance of SK-regulated genes between the tissues. Five genes (DPH2 G3BP1, G3BP2, NSUN2, and TTC17) were similarly regulated by SK1 KD in prostate and breast cancer cells (Figure 7). SK2 KD has regulated CAPZA1, ELMOD2, and HACD2 in a similar fashion across all four cell lines (Figure 7). There was no overlap between SK1 and SK2 regulated genes.

While individual genes may provide insight into altered signaling, gene-set enrichment analysis may demonstrate a bigger picture of significant pathways modulated by SK KD. Gene-set enrichment pathway analysis showed that in all cell lines SK1 KD decreased DNA conformation and replication pathways, while SK2 KD did not decrease any major pro-proliferative pathways. In contrast, there is a clear concordance in pathways upregulated in response to SK KD. SK2 KD has upregulated RAS, MAPK and small GTPase pathways, while SK1 KD has increased Wnt signaling in all four cell lines (Supplementary Tables S1–S4). In tissue specific manner, SK1 and SK2 KD individually have also increased cell motility, vesicular transport, cell motility, Rho, PI3K and serine/threonine kinase pathways. Similar to individual genes, these pathways are likely to represent the “compensatory” response of cancer cells to SKs KD and may contribute to developing resistance for anti SK therapies.

Gene-set enrichment analysis of hallmarks of cancer pathways has shown that several important pathways are upregulated in response to SK1/SK2 KD, most notably KRAS, IL2/STAT5, EMT and TNF/NFκB (SK1 KD) and EMT, G2/M and E2F (SK2 KD) (Tables 1–4). Interestingly, several well-known pathways implicated in cancer progression or apoptosis were unaffected by SK KD (such as BCL2, PARP, or many transcription factors). These pathways are also therapeutic targets and concurrent use of their inhibitors and SK inhibitors may have therapeutic advantage. For example, if one were to use SK1 inhibitor in prostate cancer, adding PI3K inhibitor may be of particular benefit as SK1 KD is upregulating PI3K pathway which may mediate resistance. Same goes for e.g., Ras pathway and SK2 in both prostate and breast cancer cells. Conversely G1/S and G2/M are both significantly inhibited by SK1 KD in breast cancer cells, therefore adding cell cycle inhibitors may be of no additional benefit.

There were no previous studies investigating transcriptome in response to SK1 KD. An opposite study was performed by Pham et al. (2014), who have transfected NIH3T3 cells with SK1 and analyzed transcriptome identifying multiple genes regulated by artificially increased SK1. There, however, may be significant difference between genes regulated by baseline SK1 activity or its absence (as in our case) and artificial SK1 overexpression. From comparison of both studies it is also clear that SK1-regulated genes are tissue and cell line specific. Angerer et al. (2018) have described transcriptome profiling of peripheral blood immune cell populations in multiple sclerosis patients before and during treatment with a sphingosine-1-phosphate receptor modulator fingolimod (also an SK1 inhibitor in higher doses) identifying *QGAP2, MYBL1*, and *PTPN12* that were consistently expressed at significantly higher transcript levels in response to continued administration of fingolimod in CD8^+^, CD4^+^ and CD19^+^ cells. Fingolimod has S1P receptor affinity at nanomolar range but requires micromolar levels to inhibit SK1 (Pchejetski et al., 2010), and it is unlikely that such levels were achieved in human patients. In addition to designing combined therapies, our data may also provide an insight into the potential side effects of therapeutic SKs inhibition. Many of the identified pathways are linked with tissue development, DNA repair, apoptosis and many other pivotal physiological processes.

As any pharmacological compounds, most SK inhibitors are not uniquely targeting SKs. Some of them have higher affinity toward SK1 (e.g., SK1-I) or SK2 (e.g., ABC294640), while others inhibit both SKs (e.g., SKI-II). They may also affect activity of other kinases. We therefore focused on using a “clean method” of SKs downregulation. It is, however, possible that siRNAs may have different effect on gene expression than pharmacological inhibitors.

## Conclusion

Through genome-wide microarray, we have identified important molecular pathways affected and not affected by SK1 and SK2 signaling. Multiple pathways such RAS, MAPK, small GTPase, Wnt and PI3K were upregulated in response to SK KD. Additionally, several well-known pathways implicated in cancer progression or apoptosis were unaffected by SK KD (such as BCL2, PARP). Co-targeting of these pathways may present a viable therapeutic option to overcome SK inhibitor resistance in prostate and breast cancer. Further signaling studies are required to confirm the individual involvement of the identified pathways.

## Author Contributions

HA developed the idea, performed the experiments, interpreted the data, edited figures and wrote the manuscript. QW provided assistance and materials. DB analyzed and interpreted data and prepared the figures. DP developed the idea, supervised experiments, interpreted the data, and wrote the manuscript. All authors reviewed the manuscript and agreed the final version of the manuscript for publication.

## Conflict of Interest Statement

The authors declare that the research was conducted in the absence of any commercial or financial relationships that could be construed as a potential conflict of interest.
