# Supplementary material for: Transcriptome-Wide Effects of Sphingosine Kinases Knockdown in Metastatic Prostate and Breast Cancer Cells: Implications for Therapeutic Targeting
Source: Front Pharmacol. 2019 Mar 27;10:303. doi: 10.3389/fphar.2019.00303 (PMC6445839; doi:10.3389/fphar.2019.00303)
Supplement: Supplementary file 5 [file Table_5.docx]

**Supplementary Table 5. Number of genes up- and downregulated in individual cell lines.**

|  | **SK1 KD upregulated** | **SK1 KD downregulated** | **SK2 KD upregulated** | **SK2 KD downregulated** |
| --- | --- | --- | --- | --- |
| **PC-3** | 1708 | 1714 | 1899 | 2050 |
| **Du145** | 881 | 877 | 1684 | 1086 |
| **MDAMB** | 114 | 100 | 50 | 22 |
| **BT457** | 1002 | 810 | 1239 | 733 |
